# Transarterial Chemoembolization Combined With Lenvatinib Plus PD-1 Inhibitor for Advanced Hepatocellular Carcinoma: A Retrospective Cohort Study

**DOI:** 10.3389/fimmu.2022.848387

**Published:** 2022-03-01

**Authors:** Mingyue Cai, Wensou Huang, Jingjun Huang, Wenbo Shi, Yongjian Guo, Licong Liang, Jingwen Zhou, Liteng Lin, Bihui Cao, Ye Chen, Juan Zhou, Kangshun Zhu

**Affiliations:** ^1^ Department of Minimally Invasive Interventional Radiology, The Second Affiliated Hospital of Guangzhou Medical University, Guangzhou, China; ^2^ Radiology Center, The Second Affiliated Hospital of Guangzhou Medical University, Guangzhou, China; ^3^ Department of Pharmacy, The Second Affiliated Hospital of Guangzhou Medical University, Guangzhou, China

**Keywords:** hepatocellular carcinoma, transarterial chemoembolization, lenvatinib, immune checkpoint inhibitor, PD-1 inhibitor, combined therapy

## Abstract

**Purpose:**

To investigate the efficacy and safety of transarterial chemoembolization (TACE) combined with lenvatinib plus PD-1 inhibitor (TACE-L-P) versus TACE combined with lenvatinib (TACE-L) for patients with advanced hepatocellular carcinoma (HCC).

**Materials and Methods:**

Data of advanced HCC patients treated with TACE-L-P (TACE-L-P group) or TACE-L (TACE-L group) from January 2019 to December 2020 were prospectively collected and retrospectively analyzed. The differences in overall survival (OS), progression-free survival (PFS), tumor responses (based on modified Response Evaluation Criteria in Solid Tumors) and adverse events (AEs) were compared between the two groups. Potential factors affecting OS and PFS were determined.

**Results:**

A total of 81 patients were included in this study. Among them, 41 received TACE-L-P and 40 received TACE-L. The patients in TACE-L-P group had prolonged OS (median, 16.9 *vs.* 12.1 months, *P*=0.009), longer PFS (median, 7.3 *vs.* 4.0 months, *P*=0.002) and higher objective response rate (56.1% *vs.* 32.5%, *P*=0.033) and disease control rate (85.4% *vs.* 62.5%, *P*=0.019) than those in TACE-L group. Multivariate analyses revealed that the treatment option of TACE-L, main portal vein invasion and extrahepatic metastasis were the independent risk factors for OS, while TACE-L and extrahepatic metastasis were the independent risk factors for PFS. In subgroup analyses, a superior survival benefit was achieved with TACE-L-P in patients with extrahepatic metastasis or tumor number >3 but not in those with main portal vein invasion. The incidence and severity of AEs in TACE-L-P group were comparable to those in TACE-L group (any grade, 92.7% *vs.* 95.0%, *P*=1.000; grade 3, 36.6% *vs.* 32.5%, *P*=0.699).

**Conclusion:**

TACE-L-P significantly improved survival over TACE-L with an acceptable safety profile in advanced HCC patients, especially those with extrahepatic metastasis or tumor number >3 but without main portal vein invasion.

## Introduction

Hepatocellular carcinoma (HCC), representing 75%-85% of primary liver cancer, is one of the most prevalent and fatal malignancies worldwide (1). Although surgical resection, ablation and liver transplantation may provide curative potential for HCC, a majority of patients are diagnosed with advanced disease which is not amenable for these approaches and thus bear a poor prognosis with an expected median survival of 6-8 months (2–4).

The multikinase inhibitors sorafenib and lenvatinib are recommended as the first-line treatment of advanced HCC (2–4) on the basis of randomized trials demonstrating longer survival with sorafenib versus placebo (5, 6) and noninferiority of lenvatinib to sorafenib (7). However, the efficacy of monotherapy with these drugs is modest, and only a small survival benefit of about 3 months can be achieved with oral sorafenib (8). In this setting, transarterial chemoembolization (TACE) has been applied to providing local disease control in patients with acceptable liver function and tumor burden. It is supposed that the antiangiogenic agents in combination with TACE may effectively offset the post-TACE hypoxia-induced angiogenesis and, therefore, provide a superior antitumor effect for HCC (9, 10). In fact, many studies have suggested improved outcomes of this combination treatment compared with the use of a single drug or TACE alone for advanced HCC (11–14). But unfortunately, there still remained limited treatment responses with unsatisfied survival prolongation (11–13).

Recently, immune checkpoint inhibitors, including programmed death 1 (PD-1) and programmed death ligand 1 (PD-L1) inhibitors, have exhibited a promising clinical benefit to advanced HCC patients (15). Although phase III trials for anti-PD-1 monotherapy failed to meet their primary survival endpoints (16, 17), the studies testing combined treatments with PD-1/PD-L1 inhibitor and antiangiogenic agent showed exciting results (18–20). In a recent phase Ib study for evaluating the combination of lenvatinib and pembrolizumab (an anti-PD-1 antibody) in first-line treatment of unresectable HCC, an objective response rate (ORR) of 46.0% per modified Response Evaluation Criteria in Solid Tumors (mRECIST) and a median overall survival (OS) of 22.0 months were achieved (20). These impressive results suggested a promising therapeutic potential of the combination of lenvatinib plus PD-1 inhibitor in patients with HCC.

Since TACE possesses a local anticancer effect and may facilitate antitumor immunity but inevitably induces post-TACE angiogenesis (10, 21), and lenvatinib has an immunomodulatory effect on tumor microenvironment besides antiangiogenesis (22, 23), combining TACE and lenvatinib plus PD-1 inhibitor (TACE-L-P) may contribute to a synergistic anticancer activity for HCC. Accordingly, we hypothesized that the comprehensive therapy of TACE-L-P would be an effective treatment strategy for advanced HCC. Thus, we conducted this retrospective study to evaluate the efficacy and safety of TACE-L-P versus TACE combined with lenvatinib (TACE-L) in the patients with advanced HCC.

## Materials and Methods

### Study Design and Patient Selection

This study was approved by our institutional review board, and written informed consent was obtained from every patient. Data of consecutive patients with advanced HCC who received TACE-L-P or TACE-L at our institution between January 2019 and December 2020 were prospectively collected and retrospectively analyzed.

The inclusion criteria for this study were as follows: 1) age between 18 and 75 years; 2) confirmed diagnosis with HCC (2, 4, 24) accompanied by macrovascular invasion and/or extrahepatic metastasis (BCLC stage C or CNLC Stage IIIa/IIIb); 3) tumor recurrence after curative resection or ablation was allowed; 4) Eastern Cooperative Oncology Group performance status (ECOG PS) of ≤1; and 5) Child-Pugh class A/B. Patients were excluded if they 1) had central nervous system metastasis; 2) had history of organ transplantation; 3) previously received TACE, hepatic arterial infusion chemotherapy (HAIC), radiotherapy or systemic therapy; 4) had other malignancies in addition to HCC; or 5) had severe medical comorbidities including severe cardiac, pulmonary, renal or coagulation dysfunction.

All laboratory test data were collected within 3 days before the initial treatment. Contrast-enhanced computed tomography (CT) or magnetic resonance imaging (MRI) was performed within 7 days before the initial treatment.

### TACE Procedure

The patients received either conventional TACE (cTACE) or drug-eluting bead TACE (DEB-TACE) according to their own choice. For cTACE, an emulsion of 5-20 mL Lipiodol (Guerbet, Paris, France) mixed with 20-60 mg pirarubicin (Hisun Pfizer Pharmaceuticals, Fuyang, China) was administered into the tumor-feeding vessels, followed by embolization with polyvinyl alcohol particles (90-500 μm; Cook, Bloomington, Indiana, USA). For DEB-TACE, CalliSpheres (Hengrui Medical, Suzhou, China) or DC Bead (Biocompatibles, Farnham, Surrey, UK) with 100-300 μm in diameter was used as the drug carrier and embolization agent. Typically, one vial of the beads was loaded with 60 mg pirarubicin. If blushed tumors were still visible after the embolization with one vial of beads, regular microspheres (8spheres, Hengrui Medical, Suzhou, China; Embosphere, Biosphere Medical, Roissy en France, France) with diameters of 100-700 μm were additionally injected (25).

During TACE, superselective catheterization was performed, and the embolization end point was blood stasis of the tumor-feeding arteries. In patients with huge or bilobar multiple lesions, in order to reduce the risk of complications, the embolization end point was not achieved in the initial TACE but in the second or third TACE session (26). In the case of arterioportal or arteriovenous fistula, the fistula would be embolized with 300-710 μm polyvinyl alcohol particles before administration of the drug-oil emulsion or drug-loaded beads.

TACE was repeated “on demand” upon the demonstration of viable tumor by follow-up CT or MRI in patients without deteriorated performance status or organ function.

### Lenvatinib and PD-1 Inhibitor Administration

Lenvatinib (Eisai, Tokyo, Japan) and PD-1 inhibitor was initiated within 7 days after the first TACE. Lenvatinib at a dose of 12 mg (bodyweight ≥60 kg) or 8 mg (bodyweight <60 kg) was orally administered once a day. The PD-1 inhibitor sintilimab (Innovent Biologics, Suzhou, China), tislelizumab (BeiGene, Shanghai, China) or camrelizumab (Hengrui Pharma, Lianyungang, China) was injected intravenously at 200 mg once every 3 weeks. The interruption and discontinuation of drug administration depended on the presence and severity of toxicities according to the drug directions.

### Follow-Up

Regular follow-up was conducted for all patients at a 3-6-week interval after the initial treatment. Each follow-up session included a detail history, physical examination, hematologic and biochemical tests, contrast-enhanced abdominal CT or MRI, chest CT, and other imaging examination if clinically indicated. The final follow-up ended on June 30, 2021.

During follow-up, the treatment of TACE-L-P or TACE-L was discontinued in cases of intolerable toxicity, progressive disease (PD) or change of treatment plan. And, the choice of the subsequent treatment, such as second-line targeted agent, PD-1 inhibitor (for the patients treated with TACE-L), radiotherapy (including iodine-125 seed brachytherapy), HAIC or best supportive care, was determined according to the results of discussion by our multidisciplinary team and the patients’ request.

### Assessments and Outcomes

OS and progression-free survival (PFS) were compared between TACE-L-P group and TACE-L group. OS was defined as the time from treatment initiation until death by any reason. PFS was defined as the time interval from treatment initiation to the first occurrence of PD or death, whichever occurred first.

Tumor responses were categorized as complete response (CR), partial response (PR), stable disease (SD) or PD according to mRECIST. Overall tumor response referred to the assessment of changes in tumor burden inside and outside the liver, while intrahepatic tumor response only included the assessment of changes in tumor burden inside the liver. ORR was defined as the percentage of patients who had a best tumor response rating of CR and PR. Disease control rate (DCR) was defined as the percentage of patients who had a best tumor response rating of CR, PR and SD.

Adverse events (AEs) related to treatments were recorded and assessed based on Common Terminology Criteria for Adverse Events (CTCAE) version 5.0. Postembolization syndrome (manifested by fever, abdominal pain, nausea, vomiting and increased white blood cell count) and transient abnormalities of liver enzyme after TACE (27, 28) were expected and would resolve within a short time, and therefore, they were not documented separately.

### Statistical Analyses

Categorical data were expressed as number of patients (percentage). Quantitative data were expressed as mean ± standard deviation and median (range) for normally and non-normally distributed variables, respectively. Categorical data between the two groups were compared using *χ* ^2^ test or Fisher’s exact test, as appropriate. Quantitative data were compared using Student’s *t*-test or Mann-Whitney U test, as appropriate. Survival curves were analyzed by Kaplan-Meier method using log-rank test. Variables with *P*<0.10 in univariate analysis were entered into a multivariate analysis using Cox regression model to identify the independent prognostic factors for OS and PFS. All statistical analyses were performed with SPSS Statistics version 22 (IBM, Armonk, New York, USA). All statistical tests were two-tailed, *P*<0.05 was considered statistically significant.

## Results

### Study Population

**Figure 1:**
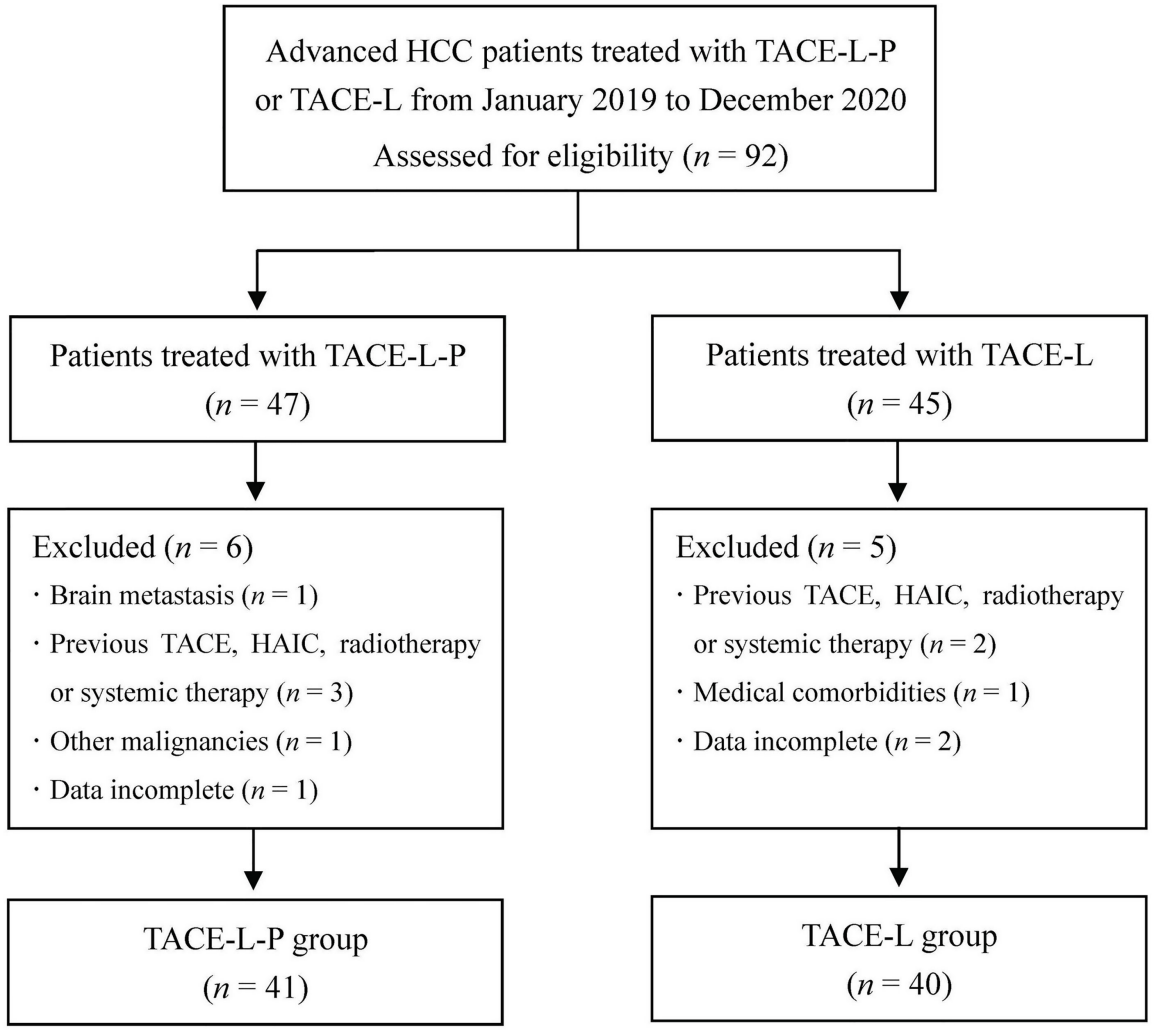
Flow diagram of patient enrollment. HCC, hepatocellular carcinoma; TACE-L-P, transarterial chemoembolization combined with lenvatinib plus PD-1 inhibitor; TACE-L, transarterial chemoembolization combined with lenvatinib; TACE, transarterial chemoembolization; HAIC, hepatic arterial infusion chemotherapy.

During the study period, 92 patients with advanced HCC who received TACE-L-P or TACE-L were screened for eligibility. Of these patients, 11 were excluded because they met the excluded criteria (**Figure 1**). Finally, 81 patients were included in this study (41 in the TACE-L-P group and 40 in the TACE-L group). Detailed baseline characteristics of the patients were summarized in **Table 1**. In both groups, about half of the patients had more than 3 intrahepatic tumors at diagnosis. The mean largest tumor size of TACE-L-P group and TACE-L group was 12.3 ± 4.8 cm and 13.6 ± 5.1 cm, respectively. Two groups were comparable in the demographic, clinical and tumor characteristics.

**Table 1:** Baseline characteristics of the patients.

Characteristic	TACE-L-P group (*n*=41)	TACE-L group (*n*=40)	*P*
Sex			0.309
Female	4 (9.8)	7 (17.5)	
Male	37 (90.2)	33 (82.5)	
Age (years)	51.9 ± 10.3	54.6 ± 11.0	0.263
ECOG PS			0.274
1	8 (19.5)	12 (30.0)	
0	33 (80.5)	28 (70.0)	
HBsAg			0.779
Positive	35 (85.4)	35 (87.5)	
Negative	6 (14.6)	5 (12.5)	
Child-Pugh class			0.309
B	4 (9.8)	7 (17.5)	
A	37 (90.2)	33 (82.5)	
AFP level (μg/L)			0.733
≥400	21 (51.2)	22 (55.0)	
<400	20 (48.8)	18 (45.0)	
PIVKA-II (mAU/ml)			0.517
≥400	27 (65.9)	29 (72.5)	
<400	14 (34.1)	11 (27.5)	
Recurrent tumor			0.362
No	35 (85.4)	31 (77.5)	
Yes	6 (14.6)	9 (22.5)	
Number of tumors			0.439
>3	23 (56.1)	19 (47.5)	
≤3	18 (43.9)	21 (52.5)	
Tumor distribution			0.939
Bilobar	28 (68.3)	27 (67.5)	
Unilobar	13 (31.7)	13 (32.5)	
Largest tumor size (cm)	12.3 ± 4.8	13.6 ± 5.1	0.218
Main portal vein invasion			0.441
Yes	15 (36.6)	18 (45.0)	
No	26 (63.4)	22 (55.0)	
Hepatic vein invasion			0.581
Yes	12 (29.3)	14 (35.0)	
No	29 (70.7)	26 (65.0)	
Extrahepatic metastasis			0.585
Yes	17 (41.5)	19 (47.5)	
No	24 (58.5)	21 (52.5)	
TACE technique			0.223
cTACE	17 (41.5)	22 (55.0)	
DEB-TACE	24 (58.5)	18 (45.0)	

Data were presented as n (%) or mean ± standard deviation. TACE-L-P, transarterial chemoembolization combined with lenvatinib plus PD-1 inhibitor; TACE-L, transarterial chemoembolization combined with lenvatinib; ECOG PS, Eastern Cooperative Oncology Group Performance Status; HBsAg, hepatitis B surface antigen; AFP, α-fetoprotein; PIVKA-II, protein induced by vitamin K absence or antagonist-II; TACE, transarterial chemoembolization; cTACE, conventional transarterial chemoembolization; DEB-TACE, drug-eluting bead transarterial chemoembolization.

The patients in TACE-L-P group underwent a total of 134 TACE procedures, with a median of 3 (range, 1-7). While the patients in TACE-L group underwent a total of 95 TACE procedures, with a median of 2 (range, 1-6). The mean duration of lenvatinib administration was 7.4 ± 3.8 (range, 1.2-15.6) months in TACE-L-P group and 4.3 ± 3.0 (range, 0.9-11.9) months in the TACE-L group (*P*<0.001). In the TACE-L-P group, the cycles of PD-1 inhibitor injection ranged from 2 to 22, with a mean of 9.9. The categories of PD-1 inhibitor the patients received were as follows: sintilimab for 30 (73.2%), tislelizumab for 6 (14.6%) and camrelizumab for 5 (12.2%).

### Survival

**Figure 2:**
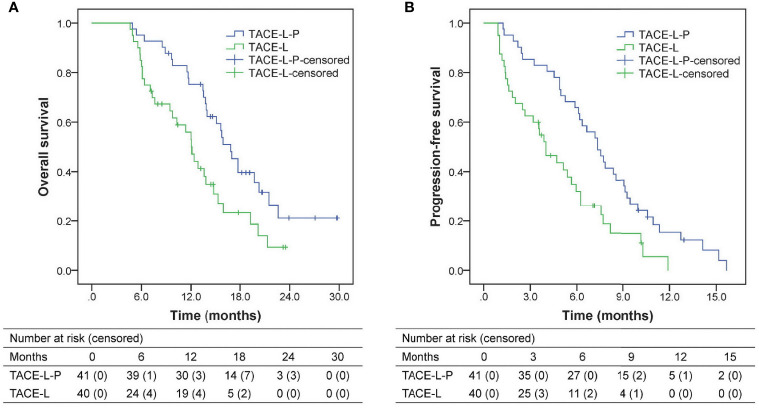
Kaplan-Meier analyses of overall survival **(A)** and progression-free survival **(B)** according to treatment groups. TACE-L-P, transarterial chemoembolization combined with lenvatinib plus PD-1 inhibitor; TACE-L, transarterial chemoembolization combined with lenvatinib.

The follow-up duration ranged from 4.6 to 29.8 months, with a median of 13.7 months. During follow-up, 27 patients (65.9%) in the TACE-L-P group and 30 patients (75.0%) in the TACE-L group died. Compared with the patients in TACE-L group, the patients in TACE-L-P group had significantly better survival outcomes (**Figure 2**). The median OS was 16.9 (95% confidence interval [CI] 14.9-18.8) months in TACE-L-P group and 12.1 (95% CI 10.7-13.5) months in TACE-L group (*P*=0.009). The median PFS was 7.3 (95% CI 6.0-8.7) months in TACE-L-P group and 4.0 (95% CI 2.7-5.3) months in TACE-L group (*P*=0.002). Additionally, the median OS and PFS of the patients treated with sintilimab were comparable to those of the patients treated with tislelizumab/camrelizumab (OS, 17.0 months [95% CI 14.4-19.6] *vs.* 16.9 months [95% CI 9.6-24.1], *P*=0.210; PFS, 7.5 months [95% CI 6.8-8.2] *vs.* 6.2 months [95% CI 4.6-7.7], *P*=0.381) in the TACE-L-P group (**Supplementary Figure 1**).

### Prognostic Factors Analysis

Based on the results of the univariate and multivariate analyses (**Table 2**), treatment option (TACE-L *vs.* TACE-L-P; hazard ratio [HR]=2.065, 95% CI 1.208-3.533, *P*=0.008), extrahepatic metastasis (present *vs.* absent; HR=2.041, 95% CI 1.183-3.520, *P*=0.010) and main portal vein invasion (yes *vs.* no; HR=1.867, 95% CI 1.089-3.200, *P*=0.023) were identified as the independent prognostic factors for OS. In addition, treatment option (HR=2.243, 95% CI 1.344-3.743, *P*=0.002) and extrahepatic metastasis (HR=2.244, 95% CI 1.365-3.689, *P*=0.001) were also identified as the independent prognostic factors for PFS.

**Table 2:** Analyses of prognostic factors for survival.

Factor	Overall survival				Progression-free survival			
	Univariate analysis		Multivariate analysis		Univariate analysis		Multivariate analysis	
	HR (95% CI)	*P*	HR (95% CI)	*P*	HR (95% CI)	*P*	HR (95% CI)	*P*
Sex								
Female/Male	1.459 (0.713-2.984)	0.301			0.951 (0.450-2.009)	0.895		
Age (years)								
<60/≥60	1.118 (0.631-1.981)	0.703			0.971 (0.581-1.626)	0.912		
ECOG PS								
1/0	1.587 (0.897-2.809)	0.113			1.271 (0.748-2.162)	0.376		
HBsAg								
Positive/Negative	1.632 (0.699-3.810)	0.257			1.045 (0.533-2.046)	0.899		
Child-Pugh class								
B/A	1.698 (0.800-3.606)	0.168			1.243 (0.631-2.448)	0.530		
AFP level (μg/L)								
≥400/<400	1.317 (0.779-2.225)	0.304			1.146 (0.718-1.828)	0.568		
PIVKA-II (mAU/mL)								
≥400/<400	1.275 (0.714-2.274)	0.411			1.387 (0.829-2.319)	0.212		
Recurrent tumor								
No/Yes	1.480 (0.723-3.030)	0.283			1.013 (0.561-1.829)	0.965		
Number of tumors								
>3/≤3	1.528 (0.893-2.615)	0.122			1.177 (0.733-1.888)	0.500		
Tumor distribution								
Bilobar/Unilobar	1.144 (0.647-2.022)	0.643			1.033 (0.623-1.711)	0.900		
Largest tumor size (cm)								
≥10/<10	1.531 (0.857-2.733)	0.150			1.388 (0.836-2.304)	0.205		
Main portal vein invasion								
Yes/No	1.638 (0.970-2.767)	0.065	1.867 (1.089-3.200)	0.023	1.025 (0.635-1.653)	0.920		
Hepatic vein invasion								
Yes/No	1.263 (0.721-2.211)	0.414			1.315 (0.783-2.206)	0.300		
Extrahepatic metastasis								
Yes/No	1.710 (1.013-2.888)	0.045	2.041 (1.183-3.520)	0.010	2.125 (1.313-3.438)	0.002	2.337 (1.430-3.820)	0.001
Treatment option								
TACE-L/TACE-L-P	1.987 (1.172-3.367)	0.011	2.065 (1.208-3.533)	0.008	2.100 (1.288-3.425)	0.003	2.312 (1.404-3.808)	0.001
TACE technique								
cTACE/DEB-TACE	1.188 (0.706-2.001)	0.517			1.311 (0.817-2.103)	0.262		

Analyses were performed using Cox proportional hazard regression model. HR, hazard ratio; CI, confidence interval; ECOG PS, Eastern Cooperative Oncology Group Performance Status; HBsAg, hepatitis B surface antigen; AFP, α-fetoprotein; PIVKA-II, protein induced by vitamin K absence or antagonist-II; TACE-L, transarterial chemoembolization combined with lenvatinib; TACE-L-P, transarterial chemoembolization combined with lenvatinib plus PD-1 inhibitor; TACE, transarterial chemoembolization; cTACE, conventional transarterial chemoembolization; DEB-TACE, drug-eluting bead transarterial chemoembolization.

**Figure 3:**
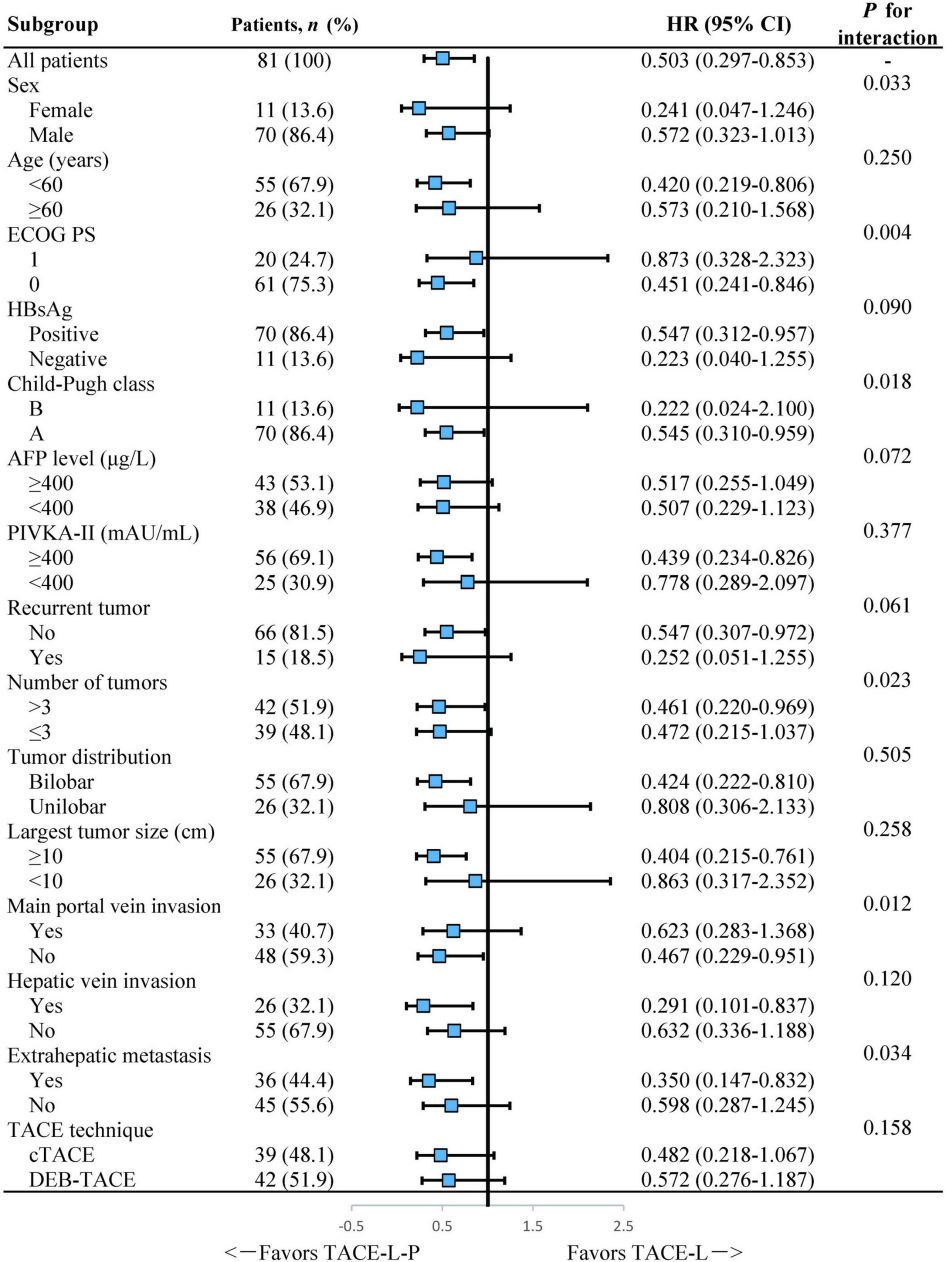
Forest plot of the subgroup analyses for overall survival. HR, hazard ratio; CI, confidence interval; ECOG PS, Eastern Cooperative Oncology Group Performance Status; HBsAg, hepatitis B surface antigen; AFP, α-fetoprotein; PIVKA-II, protein induced by vitamin K absence or antagonist-II; TACE, transarterial chemoembolization; cTACE, conventional transarterial chemoembolization; DEB-TACE, drug-eluting bead transarterial chemoembolization; TACE-L-P, transarterial chemoembolization combined with lenvatinib plus PD-1 inhibitor; TACE-L, transarterial chemoembolization combined with lenvatinib.

Subgroup analyses of factors for OS indicated that TACE-L-P treatment could provide a superior survival benefit in patients with no main portal vein invasion, tumor number >3 or extrahepatic metastasis, but failed to have a clinical benefit in patients with main portal vein invasion, tumor number ≤3 or no extrahepatic metastasis (**Figure 3**).

### Tumor Responses

**Figure 4:**
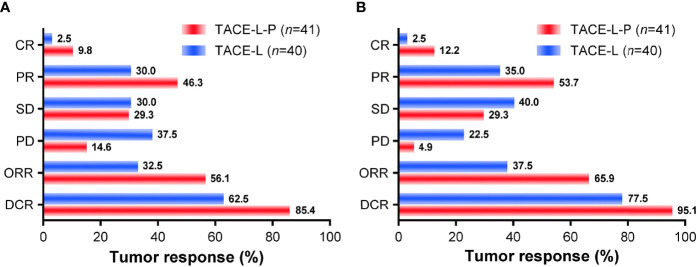
Treatment responses of overall tumor **(A)** and intrahepatic tumor **(B)** for the two groups. TACE-L-P, transarterial chemoembolization combined with lenvatinib plus PD-1 inhibitor; TACE-L, transarterial chemoembolization combined with lenvatinib; CR, complete response; PR, partial response; SD, stable disease; PD, progressive disease; ORR, objective response rate; DCR, disease control rate.

Tumor responses for patients in the two groups were shown in **Figure 4**. The ORR of both overall tumor (56.1% *vs.* 32.5%, *P*=0.033) and intrahepatic tumor (65.9% *vs.* 37.5%, *P*=0.011) was higher in the TACE-L-P group than in the TACE-L group. A higher DCR was also achieved in TACE-L-P group when compared with TACE-L group (overall tumor, 85.4% *vs.* 62.5%, *P*=0.019; intrahepatic tumor, 95.1% *vs.* 77.5%, *P*=0.021). In addition, the ORR (overall tumor, 56.7% *vs.* 54.5%, *P*=1.000; intrahepatic tumor, 66.7% *vs.* 63.6%, *P*=1.000) and DCR (overall tumor, 86.7% *vs.* 81.8%, *P*=1.000; intrahepatic tumor, 96.7% *vs.* 90.9%, *P*=0.470) of the patients treated with sintilimab were similar to those of the patients treated with tislelizumab/camrelizumab in the TACE-L-P group (**Supplementary Table 1**).

### Safety

In total, treatment-related AEs were observed in 76 of the 81 patients (93.8%), and no grade 4/5 AEs occurred (**Table 3**). The frequency and severity of AEs was similar between the TACE-L-P group and the TACE-L group (any grade, 92.7% *vs.* 95.0%, *P*=1.000; grade 3, 36.6% *vs.* 32.5%, *P*=0.699). The AEs related to TACE, including ascites, pleural effusion, inguinal hematoma and intrahepatic biliary dilatation/biloma, were mild (≤ grade 2) and occurred in 6 (14.6%) and 7 (17.5%) patients in the TACE-L-P group and the TACE-L group (*P*=0.725), respectively. The AEs related to lenvatinib and/or PD-1 inhibitor occurred in 38 (92.7%) and 37 (90.0%) patients in the TACE-L-P group and the TACE-L group (*P*=1.000), respectively. In the TACE-L-P group, the incidences of overall and grade 3 AEs in the patients treated with sintilimab were similar to those in the patients treated with tislelizumab/camrelizumab (any grade, 93.3% *vs.* 90.9%, *P*=1.000; grade 3, 36.7% *vs.* 36.4%, *P*=1.000).

**Table 3:** Treatment-related adverse events in the two groups.

Adverse events	Any grade			Grade 3		
	TACE-L-P group (*n*=41)	TACE-L group (*n*=40)	*P*	TACE-L-P group (*n*=41)	TACE-L group (*n*=40)	*P*
Total	38 (92.7)	38 (95.0)	1.000	15 (36.6)	13 (32.5)	0.699
Related to TACE	6 (14.6)	7 (17.5)	0.725	0 (0.0)	0 (0.0)	–
New ascites	3 (7.3)	2 (5.0)	1.000	0 (0.0)	0 (0.0)	–
Pleural effusion	2 (4.9)	2 (5.0)	1.000	0 (0.0)	0 (0.0)	–
Inguinal hematoma	2 (4.9)	3 (7.5)	0.977	0 (0.0)	0 (0.0)	–
Biliary injury^†^	1 (2.4)	2 (5.0)	0.983	0 (0.0)	0 (0.0)	–
Related to drug^*^	38 (92.7)	37 (92.5)	1.000	15 (36.6)	13 (32.5)	0.699
Hypertension	16 (39.0)	14 (35.0)	0.708	9 (22.0)	8 (20.0)	0.829
Weight loss	14 (34.1)	11 (27.5)	0.517	1 (2.4)	0 (0.0)	1.000
Diarrhea	13 (31.7)	13 (32.5)	0.939	0 (0.0)	0 (0.0)	–
Hand-foot syndrome	11 (26.8)	13 (32.5)	0.576	0 (0.0)	0 (0.0)	–
Fatigue	11 (26.8)	9 (22.5)	0.651	1 (2.4)	0 (0.0)	1.000
Elevated AST	11 (26.8)	8 (20.0)	0.468	0 (0.0)	1 (2.5)	0.494
Decreased appetite	10 (24.4)	9 (22.5)	0.841	1 (2.4)	1 (2.5)	1.000
Hypothyroidism	10 (24.4)	8 (20.0)	0.635	0 (0.0)	0 (0.0)	–
Elevated ALP	9 (22.0)	11 (27.5)	0.563	0 (0.0)	0 (0.0)	–
Hypoalbuminemia	9 (22.0)	8 (20.0)	0.829	0 (0.0)	0 (0.0)	–
Abdominal pain	9 (22.0)	8 (20.0)	0.829	0 (0.0)	1 (2.5)	0.494
Pruritus	9 (22.0)	4 (10.0)	0.143	0 (0.0)	0 (0.0)	–
Elevated ALT	8 (19.5)	9 (22.5)	0.741	1 (2.4)	0 (0.0)	1.000
Thrombocytopenia	8 (19.5)	8 (20.0)	0.956	1 (2.4)	2 (5.0)	0.983
Neutropenia	8 (19.5)	6 (15.0)	0.591	2 (4.9)	2 (5.0)	1.000
Proteinuria	8 (19.5)	6 (15.0)	0.591	0 (0.0)	0 (0.0)	–
Rash	8 (19.5)	4 (10.0)	0.228	0 (0.0)	0 (0.0)	–
Anemia	7 (17.1)	5 (12.5)	0.562	0 (0.0)	1 (2.5)	0.494
Lymphopenia	7 (17.1)	4 (10.0)	0.353	1 (2.4)	0 (0.0)	1.000
Elevated TBi	6 (14.6)	7 (17.5)	0.725	1 (2.4)	1 (2.5)	1.000
Nausea	6 (14.6)	6 (15.0)	0.963	0 (0.0)	0 (0.0)	–
Elevated GGT	6 (14.6)	5 (12.5)	0.779	0 (0.0)	0 (0.0)	–
Ventosity	6 (14.6)	5 (12.5)	0.779	1 (2.4)	0 (0.0)	1.000
Arthralgia	6 (14.6)	5 (12.5)	0.779	0 (0.0)	0 (0.0)	–
Vomiting	5 (12.2)	6 (15.0)	0.713	0 (0.0)	0 (0.0)	–
Gingival bleeding	5 (12.2)	6 (15.0)	0.713	0 (0.0)	0 (0.0)	–
Dysphonia	4 (9.8)	6 (15.0)	0.704	0 (0.0)	0 (0.0)	–
Edema	3 (7.3)	4 (10.0)	0.973	0 (0.0)	0 (0.0)	–
Elevated uric acid	3 (7.3)	1 (2.5)	0.626	0 (0.0)	0 (0.0)	–
Insomnia	2 (4.9)	2 (5.0)	1.000	0 (0.0)	0 (0.0)	–
Infusion reaction	2 (4.9)	–	–	0 (0.0)	–	–
Hyperglycemia	1 (2.4)	0 (0.0)	1.000	0 (0.0)	0 (0.0)	–
Pneumonitis	1 (2.4)	0 (0.0)	1.000	1 (2.4)	0 (0.0)	1.000

Data were presented as n (%). ^†^Included intrahepatic biliary dilatation and biloma; ^*^Referred to lenvatinib and/or PD-1 inhibitor. TACE-L-P, transarterial chemoembolization combined with lenvatinib plus PD-1 inhibitor; TACE-L, transarterial chemoembolization combined with lenvatinib; TACE, transarterial chemoembolization; AST, aspartate aminotransferase; ALT, alanine aminotransferase; ALP, alkaline phosphatase; TBi, total bilirubin; GGT, γ-glutamyl transpeptidase.

Treatment-related AEs led to treatment interruption, dose reduction and treatment discontinuation of lenvatinib in 22 (53.7%), 21 (51.2%) and 3 (7.3%) patients, respectively, in the TACE-L-P group, and in 20 (50.0%), 20 (50.0%) and 3 (7.5%) patients, respectively, in the TACE-L group. Treatment-related AEs led to treatment interruption and discontinuation of PD-1 inhibitor in 10 (24.4%) and 6 (14.6%) patients in the TACE-L-P group, respectively. Discontinuation of both lenvatinib and PD-1 inhibitor because of AEs occurred in only 2 patients (4.9%).

## Discussion

Our study showed that TACE-L-P conferred a significant survival benefit when compared with TACE-L in patients with advanced HCC. This finding was associated with an increase in median OS from 12.1 months to 16.9 months, which might be attributed to the higher ORR and DCR and the longer PFS achieved in patients receiving TACE-L-P rather than TACE-L. Multivariate analyses also revealed that combining PD-1 inhibitor on the basis of TACE-L was an independent predictor for prolonged OS and PFS. These results indicated that the triple combination treatment of TACE-L-P might be a superior treatment option in advanced HCC patients. The reasons might be as follows: 1) TACE lead to an extensive local necrosis of the tumor and may subsequently elicit anticancer immune responses that may be further boosted with PD-1 inhibitors (10, 21). 2) Lenvatinib is a multikinase inhibitor with antiproliferative and antiangiogenitic activities (22), which may counteract the hypoxia-induced angiogenesis after TACE (9, 10) and can regulate the tumor immune microenvironment and enhance immune response of PD-1 inhibitor in HCC (22, 23). Therefore, the combination of TACE, lenvatinib and PD-1 inhibitor may bring about a synergistic antitumor activity, contributing to improved clinical outcomes in advanced HCC patients.

Previous studies (29, 30) have assessed the combination of TACE, lenvatinib and PD-1 inhibitor for patients with unresectable HCC and reported a PFS of 11.4-13.3 months and an OS of 23.6-24.0 months, which seemed much longer than those for the patients treated with TACE-L-P in our study. However, it was worth noting that these studies enrolled a large proportion (25.0%-54.5%) of patients with HCC at BCLC stage B, who were expected to achieve better outcomes than those with BCLC stage C HCC in the present study. Additionally, the heavier tumor burden the patients borne in our study (largest tumor size of 12.3 ± 4.8 cm and a considerable proportion of patients with >3 intrahepatic tumors, bilobar tumor distribution, main portal vein invasion or extrahepatic metastasis) might also lead to a limited survival benefit of treatment. But then again, compared with TACE-L, TACE-L-P did provide a significant improvement in survival for the HCC patients with advanced disease.

In our study, the presence of main portal vein invasion or extrahepatic metastasis was identified as the independent risk factor for survival. These results were consistent with previous studies (31–34) and further confirmed that main portal vein invasion or extrahepatic spread had a profound adverse effect on prognosis in HCC patients. More notably, in subgroup analyses, a prolonged OS was observed with the treatment of TACE-L-P not in patients with main portal vein invasion but in those without main portal vein invasion, which implied that TACE-L-P might be better employed for HCC patients before the main portal trunk was involved so that an improved survival could be achieved. Furthermore, subgroup analyses also showed that TACE-L-P provided a better OS than TACE-L in the patients with extrahepatic metastasis or tumor number >3 but not in those with no extrahepatic metastasis or tumor number ≤3. The reasons might be that TACE exerted its antitumor property mainly by controlling intrahepatic lesions rather than extrahepatic metastases (9) and its effect on multiple tumors was also limited (35). Thus, a treatment strategy combining TACE with a more potent systemic therapy was urgently needed for patients with extrahepatic metastasis or multiple tumors. Our results revealed the necessity of the additional treatment with PD-1 inhibitor to TACE-L for such patients.

In our study, all the AEs with the combination of TACE and lenvatinib with/without PD-1 inhibitor were manageable and consistent with previously reported data on each individual treatment (7, 10, 12, 36). There were no new or unexpected AEs observed. Additionally, the incidence and severity of AEs in TACE-L-P group were comparable to those in TACE-L group. These results suggested that both the treatments of TACE-L-P and TACE-L were tolerable and combining PD-1 inhibitor with TACE-L did not significantly increase the risk of AEs compared with TACE-L, which revealed an acceptable safety profile of TACE-L-P.

In the present study, three different PD-1 inhibitors were used for the treatment of patients in TACE-L-P group. Although our results suggested that the tumor responses, survival and incidence of AEs in the patients treated with sintilimab were similar to those in the patients treated with tislelizumab/camrelizumab, the inconformity of treatment with these PD-1 inhibitors and its potential effects on treatment outcomes remained to be concerned. Additionally, our study had some other limitations. First, this study was a retrospective study, and the treatment option was individually determined based on the preference of the attending physician and the patient, which inevitably resulted in selection bias. Second, the sample size of this study was limited. The results of subgroup analyses should be cautiously interpreted. Consequently, validation of our findings by further randomized trials is necessary.

In conclusion, our study showed safety and promising outcomes with the treatment of TACE-L-P in patients with advanced HCC. These patients could benefit from TACE-L-P and had markedly better treatment responses and improved survival in comparison with TACE-L. These findings need to be confirmed in large sample, prospective randomized controlled trials.

## Data Availability Statement

The raw data supporting the conclusions of this article will be made available by the authors, without undue reservation.

## Ethics Statement

The studies involving human participants were reviewed and approved by the Clinical Research and Application Ethics Committee of the Second Affiliated Hospital of Guangzhou Medical University. The patients/participants provided their written informed consent to participate in this study.

## Author Contributions

MC, WH, and JH conceived and designed the study with supervision from KZ. MC, WH, YG, JWZ, and KZ provided the provision of study materials or patients. WS, LCL, LTL, BC, YC, and JZ collected and assembled the data. MC, JH, and WS performed or supervised analyses. MC, WH, and KZ interpreted the results. MC, JH, and KZ wrote the manuscript. All authors reviewed the manuscript and approved the final version.

## Funding

This work was supported by the National Natural Science Foundation of China (81873920, 82001930, 82001929), the Science and Technology Project of Guangzhou, China (202002030135, 202102010082), the High-Level University Clinical Research Promotion Program of Guangzhou Medical University (B185004019), and the Doctoral Scientific Research Fund of the Second Affiliated Hospital of Guangzhou Medical University (010G271073).

## Conflict of Interest

The authors declare that the research was conducted in the absence of any commercial or financial relationships that could be construed as a potential conflict of interest.

## Publisher’s Note

All claims expressed in this article are solely those of the authors and do not necessarily represent those of their affiliated organizations, or those of the publisher, the editors and the reviewers. Any product that may be evaluated in this article, or claim that may be made by its manufacturer, is not guaranteed or endorsed by the publisher.
