# Supplementary material for: Transarterial Chemoembolization Combined With Lenvatinib Plus PD-1 Inhibitor for Advanced Hepatocellular Carcinoma: A Retrospective Cohort Study
Source: Front Immunol. 2022 Mar 1;13:848387. doi: 10.3389/fimmu.2022.848387 (PMC8921060; doi:10.3389/fimmu.2022.848387)
Supplement: Supplementary file 1 [file DataSheet_1.docx]

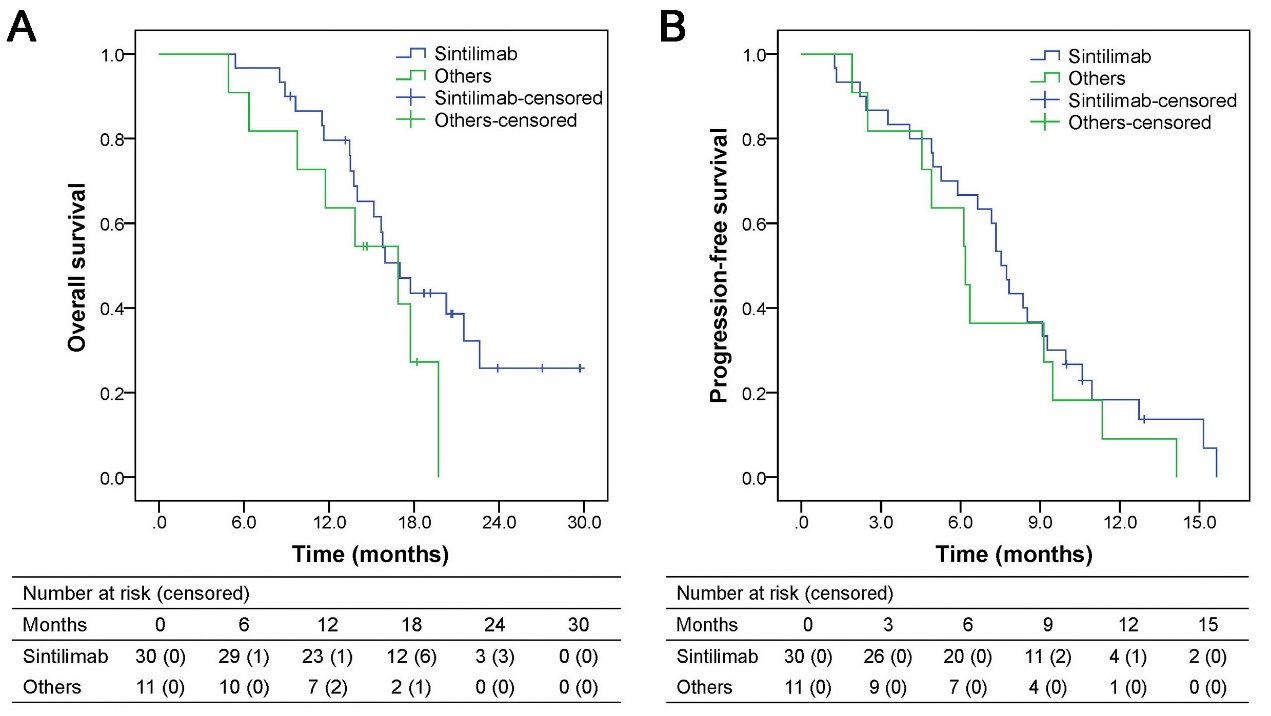


**SUPPLEMENTARY FIGURE 1 |** Kaplan-Meier analyses of overall survival **(A)** and progression-free survival **(B)** according to different PD-1 inhibitors in the patients who received triple combination treatment. Others referred to the other PD-1 inhibitors (tislelizumab and camrelizumab).
