# Supplementary material for: Transarterial Chemoembolization Combined With Lenvatinib Plus PD-1 Inhibitor for Advanced Hepatocellular Carcinoma: A Retrospective Cohort Study
Source: Front Immunol. 2022 Mar 1;13:848387. doi: 10.3389/fimmu.2022.848387 (PMC8921060; doi:10.3389/fimmu.2022.848387)
Supplement: Supplementary file 2 [file Table_1.docx]

**SUPPLEMENTARY TABLE 1 |** Tumor responses for the patients who received the triple combination treatment.

| Response | Sintilimab  (*n*=30) | Others^*^  (*n*=11) | *P* |
| --- | --- | --- | --- |
| Overall tumor |  |  |  |
| CR, *n* (%) | 3 (10.0) | 1 (9.1) |  |
| PR, *n* (%) | 14 (46.7) | 5 (45.5) |  |
| SD, *n* (%) | 9 (30.0) | 3 (27.3) |  |
| PD, *n* (%) | 4 (13.3) | 2 (18.2) |  |
| ORR, % | 56.7 | 54.5 | 1.000 |
| DCR, % | 86.7 | 81.8 | 1.000 |
| Intrahepatic tumor |  |  |  |
| CR, *n* (%) | 4 (13.3) | 1 (9.1) |  |
| PR, *n* (%) | 16 (53.3) | 6 (54.5) |  |
| SD, *n* (%) | 9 (30.0) | 3 (27.3) |  |
| PD, *n* (%) | 1 (3.3) | 1 (9.1) |  |
| ORR, % | 66.7 | 63.6 | 1.000 |
| DCR, % | 96.7 | 90.9 | 0.470 |

*^*^Referred to tislelizumab and camrelizumab. CR, complete response; PR, partial response; SD, stable disease; PD, progressive disease; ORR, objective response rate; DCR, disease control rate.*
